# The mediating effects of childhood neglect on the association between schizotypal and autistic personality traits and depression in a non-clinical sample

**DOI:** 10.1186/s12888-017-1510-0

**Published:** 2017-10-25

**Authors:** Jianbo Liu, Jingbo Gong, Guanghui Nie, Yuqiong He, Bo Xiao, Yanmei Shen, Xuerong Luo

**Affiliations:** 1 Mental Health Institute of The Second Xiangya Hospital and Key Laboratory of Psychiatry and Mental Health of Hunan Province, The Central South University, Changsha, 410000 China; 2 Department of Applied Psychology, Traditional Chinese Medicine University of Hunan, Changsha, 410208 China; 3 School of Public Health, Guangxi Medical University, Nanning, 530000 China

**Keywords:** APT, SPT, Neglect, Depression, Mediating factor

## Abstract

**Background:**

Autistic personality traits (APT) and schizotypal personality traits (SPT) are associated with depression. However, mediating factors within these relationships have not yet been explored. Thus, the focus of the current study was to examine the effects of childhood neglect on the relationship between APT/SPT and depression.

**Methods:**

This cross-sectional study was conducted on first-year students (*N* = 2469) at Hunan University of Chinese Medicine and Hengyang Normal College (Changsha, China). Participants completed surveys on APT, SPT, childhood neglect, abuse and depression.

**Results:**

Through correlational analyses, APT and SPT traits were positively correlated with childhood neglect and depression (*p* < 0.05). In a hierarchical regression analysis, among types of childhood maltreatment, emotional neglect (β = 0.112, *p* < 0.001) and physical neglect (β = 0.105, *p* < 0.001) were the strongest predictors of depression. Childhood neglect did not account for the relationships between APT/SPT and depression. Further analysis found that childhood neglect mediated the relationship between SPT and depression but not APT and depression.

**Conclusions:**

Among types of childhood maltreatment, neglect was the strongest predicting factor for depression. Neglect did not account for the relationship between APT/SPT and depression but was a strong mediating factor between SPT and depression.

## Background

According to the Diagnostic and Statistical Manual, autism spectrum disorder (ASD) and schizophrenia spectrum disorder (SPD) are categorized into two different neuronal development disorders. However, studies found overlap between ASD and SPD, including accompanying depression symptoms [1, 2]. Depression has been reported as the highest comorbid symptoms with ASD [3]. Matson et al. [3] reported that 30% of those diagnosed with ASD are also diagnosed with depression [4]. Onwuameze et al. [2] reported rates of depression that ranged from 18%–41% in the different assessment periods for SPD. Prior research has shown that ASD and SPD are both correlated with dysregulation of the hypothalamic-pituitary-adrenal (HPA) axis, which may also be linked to depression [5, 6]. Additionally, some genes associated with an increased risk of SPD and APD also increase the risk of suffering from depression. For example, MTHFR gene C667T polymorphism increases the risk of autism [7] and also increases the risk of depression [8]. Similarly, the COMT gene rs4680 increases the risk of SPD [9] and also increases the risk of depression [10].

Recent research found that autistic personality traits (APT) and schizotypal personality traits (SPT) not only exist in ASD and SPD but also as subclinical psychiatric symptoms in the general population. APT are characterized by social and communication issues as well as restricted, repetitive and stereotyped patterns of behavior, interests and activities [11]. SPT include magical thinking, odd or bizarre behavior, and abnormal perceptual experiences and are considered one of the possible traits of a latent vulnerability to psychosis (schizotypy traits) [12].

Research has shown an overlap between APT and SPT [13] and found that significant positive correlations exist between APT/SPT and depression symptoms [14–16]. However, the confounding factors between APT/SPT and depression have not yet been identified. Therefore, the current study focused on the influence of several factors to better understand the association between APT/SPT and depression.

Childhood neglect includes both emotional and physical neglect by guardians, causing harm to a child’s health and/or development [17]. The prevalence of childhood neglect is greater than any other childhood maltreatment [18]. Neglect not only causes economic losses [19], but also increases the risk of criminality [20] and psychiatric illnesses such as depression [21]. Moreover, one study found that depression severity in orphans is related more strongly to neglect than abuse [22]. Interestingly, MRI studies have found that childhood neglect is associated with reduced brain volume in the regions of the brain responsible for memory function [23].

The risk of childhood abuse and neglect is greater among children and adolescents with disabilities [24–26]. Since APT and SPT are often diagnosed in childhood [27, 28], individuals with APT and SPT may experience more childhood neglect compared to healthy individuals due to communication problems and limited social skills [11]. In fact, research has found an association between childhood neglect and SPT. For example, Berenbaum et al. [29] found that neglect was most strongly associated with schizotypal symptoms among different forms of childhood maltreatment. However, to date, no studies have found an association between neglect and APT. According to a study by Sullivan et al. [25], people diagnosed with ASDs are at an increased risk of childhood neglect. Therefore, it is likely that there is an increased risk of neglect in participants with APT.

Based on the prior research described above, we formulated three hypotheses: (1) that a strong link would exist between APT/SPT and neglect, (2) that of all childhood maltreatments, childhood neglect would be the strongest predicting factor for depression and (3) that childhood neglect would be a mediating factor between APT/SPT and depression.

## Method

### Participants

A paper-and-pencil survey was administered to 2757 first-year college students (758 males and 1989 females) studying clinical medicine, nursing, psychology and education at Hunan University of Chinese Medicine and Hengyang Normal College (Changsha and Hengyang, China). The survey was administered during school hours with no time constraints. Surveys with more than 10% missing data were omitted from the analysis. The final analysis was obtained from a sample size of 2469 participants (684 males and 1785 females). The average age of the participants was 18.76 ± 1.12 years. There was no significant age difference between males and females. The prevalence of childhood abuse was evaluated according to the methodology of Bernstein (1997) [30]. The Ethics Committee of the Second Hospital of Hunan University of Chinese Medicine approved the study. Table 1 displays the demographic characteristics of the participants.

**Table 1:** Demographic characteristics of the students in the study (*N* = 2469)

Variables		Frequency (%)
Gender		
	Male	684 (27.7%)
	Female	1785 (72.3%)
Family status		
	Only-child family	819 (33.17%)
	Not only-child family	1650 (66.83%)
Parents’ marital status		
	Completed families	1936 (78.41%)
	Unmarried	529 (21.43%)
	Others	4 (0.16%)
Monthly family income per person		
	< 1000 yuan	825 (33.41%)
	1000–1449 yuan	467 (18.91%)
	≥ 1500 yuan	1177 (47.68%)
The prevalence of childhood trauma		
	Emotional abuse	128 (5.18%)
	Physical abuse	229 (9.28%)
	Sexual abuse	237 (9.60%)
	Emotional neglect	341 (13.81%)
	Physical neglect	495 (20.05%)

Note: “Unmarried” includes divorced and widowed, “Other” includes missing data

### Measures

#### Autism spectrum quotient (AQ)

The AQ is a 50-item self-rated questionnaire measuring autistic personality trait in intellectually normal adults [31]. Each item on the AQ is scored on a 4-point scoring system ranging from “strongly agree” to “strongly disagree”. Each item was rated as “1” or “0”; hence 50 was the highest attainable AQ total score [31]. Higher scores indicated a greater number of autistic personality traits. In Mainland China, the AQ total has an internal consistency of 0.806 and test-retest reliability has been recorded as 0.89 (Pearson’s r correlation coefficient) [32].

### Schizotypal personality questionnaire (SPQ)

The SPQ is a 74-item self-rated questionnaire measuring SPD (positive symptoms, negative symptoms and disorganized symptoms) [33]. The SPQ is a dichotomous item questionnaire (yes or no) and each item is scored as 1 (yes) or 0 (no); hence 74 is the highest SPQ score and higher scores indicate severe SPD. A Chinese Taiwan version of the SPQ has been developed [34]. The internal consistency of the SPQ total score is 0.90 in adults and 0.93 in adolescents.

### Childhood trauma questionnaire-short form (CTQ-SF)

The CTQ-SF [35] is a 28-item self-rated questionnaire that measures the incidence of five types of childhood maltreatment: emotional abuse (EA), physical abuse (PA), sexual abuse (SA), emotional neglect (EN) and physical neglect (PN).

The CTQ is the most commonly used questionnaire to measure incidence of childhood abuse and neglect. The CTQ has proved to be a reliable and valid screening tool [35]. The items are rated on a 5-point scale ranging from 1 (“never true”) to 5 (“very often true”). The score for each trauma therefore ranges from 5 to 25 with higher scores indicating greater trauma severity.

A Chinese version of CTQ-SF has been developed [36]. The Cronbach’s alpha coefficients for the subscales of the Chinese version of the CTQ-SF range from 0.51 to 0.71 and the test-retest correlations range from 0.68 to 0.82, except for physical neglect which has a test-retest correlation of 0.43.

### The Zung self-rating depression scale (SDS)

The SDS, designed by Zung in 1965, is a 20-item self-report questionnaire that measures depressive symptoms [37]. The SDS is a suitable and common questionnaire that measures depression symptoms among adults. Higher scores indicate greater severity of depression symptoms. The correlation between each item and SDS total was greater than 0.5 in Chinese college students [38].

### Statistical analysis

Data analyses were conducted using SPSS version 21 and AMOS version 22 statistical software (IBM, Armonk, NY). Correlation analyses were performed to test the relationships among SPQ, AQ, abuse, neglect and depression. A hierarchical regression model was used to test the predicting strength of APT/SPT total on depression after controlling for gender, abuse and neglect. A mediation model with bootstrapping method (a bootstrap sample of 2000 was specified) was used to assess indirect effects of neglect [39]. The indirect model was used since the bootstrapping confidence intervals did not include zero [40]. In addition, the Chi-squared test (*p >* 0.05), the Goodness-of-Fit Index (GFI, >0.90), the Adjusted Goodness-of-Fit Index (AGFI, >0.90), the Root-Mean-Square Error of Approximation (RMSEA, <0.06) and the Comparative Fit Index (CFI, >0.95) were used to evaluate the mediation model [41, 42].

## Results

### Descriptive statistics and correlation

Correlation analyses found that AQ total, SPQ total, abuse and neglect were positively related to depression (all *p*-values <0.01), and that AQ total and SPQ total were positively related to neglect and abuse (*p* < 0.01) (Table 2).

**Table 2:** Descriptive statistics and the correlation for study measure (*n* = 2469)

	SPQ total	AQ total	EA	PA	SA	EN	PN	Depression
SPQ total	1.00							
AQ total	.419**	1						
EA	.376**	.143**	1					
PA	.238**	.085**	.578**	1				
SA	.267**	.090**	.523**	.617**	1			
EN	.197**	.114**	.449**	.431**	.321**	1		
PN	.236**	.085**	.469**	.424**	.397**	.591**	1.00	
Depression	.484**	.337**	.342**	.262**	.287**	.305**	.317**	1
Mean (total)	25.40	22.18	7.28	6.21	5.76	9.56	7.54	37.66
SD (total)	12.79	5.32	2.63	2.47	2.09	4.15	2.93	7.95
Mean (male)	27.48	22.22	7.48	6.95	6.41	10.14	8.15	38.17
SD (male)	14.17	4.88	2.94	3.27	2.98	4.52	3.33	8.52
Mean (female)	24.61	22.16	7.20	5.93	5.50	9.33	7.30	37.46
SD (female)	12.13	5.48	2.49	2.03	1.55	3.98	2.73	7.71

**<0.01 *EA* emotional abuse; *PA* physical abuse; *SA* sexual abuse; *EN* emotional neglect; *PN* physical neglect

### APT/SPT predicted depression after controlling for gender, abuse and neglect

The hierarchical regression model was significant after gender was entered in step 1 and SPQ total was entered in step 2 (△R^2^ = 0.233, F_1, 2466_ = 748.606, *p* < 0.001). SPQ total was an independent predictor for depression (β = 0.485, *p* < 0.001) (see Table 3). The AQ total was entered in step 3 and this model was also significant (△R^2^ = 0.022, F_1, 2465_ = 71.919, *p* < 0.001). SPQ total (β = 0.416, *p* < 0.001) and AQ total (β = 0.162, *p* < 0.001) were independent predictors for depression (see Table 3).

**Table 3:** Hierarchical regression model of predictors of depression (*n* = 2469)

	Adjusted R^2^	∆R^2^	β	*P*
Step 1	0.001	0.002		
Gender			−0.04	0.047
Step 2	0.233	0.233		
Gender			0.009	0.629
SPQ total			0.485	<0.001
Step 3	0.255	0.022		
Gender			0.003	0.882
SPQ total			0.416	<0.001
AQ total			0.162	<0.001
Step 4	0.296	0.042		
gender			0.028	0.110
SPQ total			0.336	<0.001
AQ total			0.167	<0.001
EA			0.115	<0.001
PA			0.045	0.053
SA			0.100	<0.001
Step 5	0.321	0.025		
Gender			0.037	0.032
SPQ total			0.33	<0.001
AQ Total			0.162	<0.001
EA			0.051	0.026
PA			0.001	0.974
SA			0.087	<0.001
EN			0.112	<0.001
PN			0.105	<0.001

*Β* standardized regression coefficient; *EA* emotional abuse; *PA* physical abuse; *SA* sexual abuse; EN emotional neglect, *PN* physical neglect

At step 4, variables of abuse were entered and the model was again significant (△R^2^ = 0.042, F_3, 3462_ = 48.617, *p* < 0.001). SPQ total (β = 0.336, *p* < 0.001), AQ total (β = 0.167, *p* < 0.001), emotional abuse (β = 0.115, *p* < 0.001) and sexual abuse (β = 0.1, p < 0.001) were identified as independent predictors for depression (see Table 3).

At step 5, neglect variables were entered and this model was also significant (△R^2^ = 0.025, F_2, 2460_ = 45.967, *p* < 0.001). Neglect did not alter the relationship between AQ/SPQ and depression, indicating that neglect does not account for the relationship between AQ/SPQ and depression. However, SPQ total (β = 0.33, *p* < 0.001) and AQ total (β = 0.162, *p* < 0.001) remained independent predictors for depression.

Neglect (emotional neglect: β = 0.112, *p* < 0.001; physical neglect: β = 0.105, *p* < 0.001) was the strongest predictor of depression among all childhood maltreatments (see Table 3).

### Mediation analysis between SPQ/AQ and depression

One objective of the current research was to test the hypothesis that childhood neglect mediates the relationship between autistic or schizotypal personality traits and depression. However, there was no relationship between AQ total and neglect (*p* > 0.05) thus indicating that childhood neglect is not a mediator between autistic personality traits and depression. Therefore we excluded the relationship between AQ total and neglect in further analyses.

**Fig. 1:**
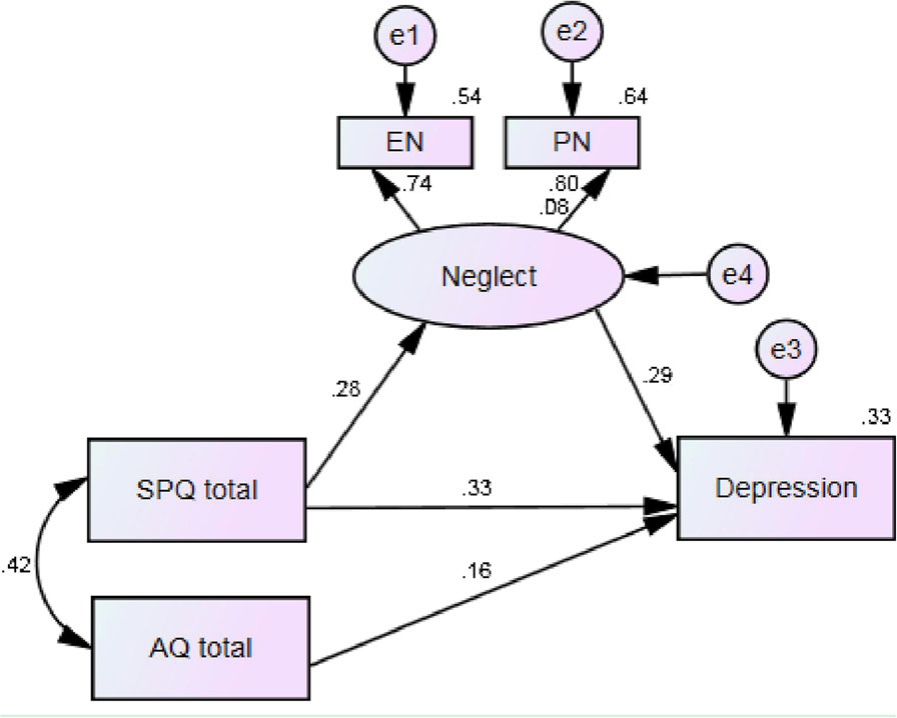
Neglect mediates the relationship between SPQ and depression

The estimated standardized regression coefficients for the effect of AQ total, SPQ total and neglect on depression were 0.16, 0.33 and 0.29, respectively (all *p*-values <0.001) and the estimated standardized regression coefficient for the effect of SPQ total on neglect was 0.28 (*p* < 0.001) (See Fig. 1).

The bootstrapping confidence intervals of all paths, which test the mediating factor of childhood neglect between SPT and depression, did not include zero (see Table 4) thus indicating that childhood neglect is a significant mediator in the relationship between SPT and depression.

**Table 4:** Mediation analysis examining the indirect effect of neglect between SPQ total and depression

			Bootstrapping			
	Standardized estimate	SE	Bias-corrected		Percentile	
			95% CI		95% CI	
			Lower	Upper	Lower	Upper
SPQ total → depression			Standardized total effect			
	0.416	0.019	0.379	0.454	0.377	0.452
SPQ total → depression			Standardized indirect effect			
	0.082	0.010	0.064	0.103	0.064	0.102
SPQ total → depression			Standardized direct effect			
	0.335	0.020	0.294	0.374	0.293	0.372

The fit indices (GFI = 0.998, AGFI = 0.992, RMSEA = 0.031 and CFI = 0.997) indicated satisfactory model fits. However, although the Chi-squared (χ^2^) test reported a *p* < 0.05 indicating that the model was not acceptable, the χ^2^ test is sensitive to large sample sizes and so other suitable statistics should be considered.

## Discussion

In this study we explored factors that impact the relationship between APT/SPT and depression in a large sample of college students. To our knowledge, this is the first study to examine childhood neglect as a mediating factor. The results will add to the current understanding of the relationship between APT/SPT and depression.

The current study found that APT and SPT are independent predictors of depression symptoms, which is in agreement with previous literature. For example, Fonseca-Pedrero et al. [14] found that SPT and subclinical depressive symptoms were highly overlapping phenomena among the nonclinical adolescent population. Shi et al. [43] found that participants with higher SPQ scores (≥36) also scored higher on depressive symptoms compared to those with low SPQ scores (≤10). Xu et al. [44] found that APT also positively predicted depression symptoms.

Additionally, in adult patients with mood disorders, Matsuo et al. [45] found that APT were associated with depression symptom severity. As participants with autistic traits cannot easily adapt to the changing environment, they are perhaps more vulnerable to depression symptoms [46]. For participants with schizotypal traits, many prior studies have found that positive and negative SPT symptoms increase depression symptoms [14]. Moreover, SPT can play a detrimental role in cognitive function [47], thus leading to an increased depression [48].

HPA-axis dysregulation has previously been reported in clinical patients with SPD and APD [5, 6]. A dysregulation of the HPA-axis can lead to a cortisol abnormality [6], which can in turn increase depression [6]. Therefore, the underlying cause of depression symptoms associated with APT and SPT may be similar.

From a genetic perspective, Gong et al. [49] reported that HTR2A gene T102C polymorphism is associated with autistic-like behavior in healthy individuals; Jokela et al. [50] also found that HTR2A gene T102C polymorphism may affect depression symptoms through gene-environment interactions. It is therefore possible that APT and depression symptoms may have a common underlying genetic cause. Similarly, COMT Val158Met significantly influences schizotypal trait scores among healthy people [51] and plays a role in the susceptibility to depressive symptoms among postmenopausal women [52].

Consistent with our hypothesis stated earlier in this paper and in previous research, among all childhood maltreatments, neglect was the strongest predictor for depressive symptoms. Hermenau et al. [22] found that in a sample of orphans the experience of neglect (but not abuse) was correlated with depression severity. A recent meta-analysis also reported that neglect is a stronger predictor of depression than either sexual or physical abuse [53].

Interestingly, recent research found that neglect (as an childhood stress) can affect gene expression, DNA transcription and translation [54, 55], which may in turn affect some genes related to depression, as described earlier.

After controlling for neglect, the relationships between APT/SPT and depression remained significant thus indicating that neglect does not account for these relationships. However, upon further analysis, we found that childhood neglect mediated the relationship between SPT and depression.

To our knowledge, this is the first report demonstrating how the link between SPT and depression is mediated by neglect. In agreement with previous research [29], the current study found that SPT were positively correlated with childhood neglect. In fact, it has previously been shown that a variety of disabilities may increase the susceptibility of childhood abuse and neglect [24, 26]. In addition, these results also suggest that the incidence of neglect must be factored into the treatment of children with SPT.

Childhood neglect can increase depression [56]. While our findings may give some insight into the relationship between SPT and depression, other factors such as heredity and the interaction between environment and heredity should be further explored [57–59].

Although there was a trend for APT to be positively correlated with neglect in the correlational analysis, there was no relationship between APT and neglect in the mediation analysis. One possible explanation for this discrepancy is the difference in statistical methods. The correlation analysis (a single factor analysis) was relatively unstable compared to mediation analysis.

While the current study provides significant insight into the relationships between ATP/SPT and depression, there are some limitations that must be considered. First, whether the results of the current study are applicable to a clinical population is unknown, since all participants were healthy college students. Thus, future research should include clinically diagnosed SPD and ASD patients.

Second, the current study only explored childhood mental factors affecting the relationships between APT/SPT and depression. Future studies should include genetic effects on the relationships between APT/SPT and depression [10] as well as the effects of the interaction between genetics and environment.

Third, since the participants were from medical and general colleges that tend to have more female than male students, there was a large discrepancy between the number of male and female participants in the current study.

Fourth, data were collected through self-report questionnaires, which are prone to bias since participants wish to provide socially desirable responses. Specifically, recall bias is inherent to questions on the CTQ relating to the recall of childhood traumas.

Fifth, the Zung self-rating depression scale is well-validated in the measurement of depression symptoms [37]. However, the validity of the questionnaire in the measurement of depression trait is unclear. Therefore, future research should use a depression trait scale to further explore the association between ATP/SPT and depression.

Sixth, SPT and depression can confound one another. For example, anhedonia-like characteristics are shared between SPT and depression and as such these factors are difficult to dissociate.

Seventh, this study is a cross-section study, and therefore, causal relationships can not be determined.

Eighth, relational data, such as general psychopathology in the family, which may increase risk for general psychopathology were not gathered in the current study. In future studies, we will further test the mediating effects of neglect in the relationship between SPT and depression controlling for psychopathology in the family.

Lastly, although CTQ is a commonly accepted scale for assessing childhood abuse and neglect [35], some recent studies have found that CTQ may lack structural invariance in cross-cultural adaptations [60].

## Conclusions

To our knowledge, this is the first study to examine childhood neglect as a mediating factor in the relationship between APT/SPT and depression. Among types of childhood maltreatment, neglect was the strongest predicting factor for depression. Neglect did not account for the relationship between APT/SPT and depression but was a strong mediating factor between SPT and depression.

## Abbreviations

APT: Autistic personality traits
ASD: Autism spectrum disorder
HPA: Hypothalamic-pituitary-adrenal
SPD: Schizophrenia spectrum disorder
SPT: Schizotypal personality traits
